# Zero Fluoroscopy Ablation of Arrhythmias in Patients With Congenital Heart Disease

**DOI:** 10.1002/joa3.70225

**Published:** 2025-11-17

**Authors:** Shailendra Upadhyay, Jenna Schermerhorn, Whitney Fairchild, Jamie Bopp, Irfan Warsy

**Affiliations:** ^1^ School of Medicine University of Connecticut Farmington Connecticut USA; ^2^ Connecticut Children's Hartford Connecticut USA; ^3^ Children's Mercy Hospital Kansas City Missouri USA

**Keywords:** arrhythmia, congenital heart disease, electroanatomic mapping, fluoroscopy, radiofrequency ablation

## Abstract

**Aims:**

Radiation‐free catheter ablation is feasible with modern electroanatomic mapping systems. We aimed to evaluate the feasibility, safety, and outcomes of non‐fluoroscopic ablation (NFA) in patients with congenital heart disease (CHD).

**Methods:**

We retrospectively reviewed CHD patients who underwent NFA between November 2016 and January 2025. All procedures were performed using the CARTO 3D electroanatomic mapping system. Atrial, ventricular, and aortic geometries were reconstructed as needed. Catheter navigation and sheath placement were guided without fluoroscopy; intracardiac echocardiography was used selectively.

**Results:**

Forty‐two patients (23 females) with CHD underwent NFA. The median age was 14 years (range 4–56), and median weight was 55 kg (range 19–145). Twenty‐one patients had mild, 16 moderate complexity and 5 great complexity CHD. Arrhythmia mechanisms included AVNRT (14%), manifest WPW (21%), high‐risk WPW without SVT (5%), concealed pathway AVRT (26%), AFL (12%), AT (14%), and VT (7%). Two patients had both AVNRT and AVRT. Acute success was achieved in all cases without fluoroscopy or acute complications. Over a median 48‐month follow‐up, three patients had recurrences: one with WPW and Ebstein anomaly, one with ASD/PLSVC and concealed pathway, and one with dual arrhythmia substrates.

**Conclusion:**

Zero‐fluoroscopy ablation of arrhythmias in select patients with mild moderate or great complexity CHD is feasible, safe, and effective, offering high acute success and low recurrence while eliminating radiation exposure.

## Graphical abstract

Zero‐fluoroscopy radiofrequency ablation using 3D electroanatomic mapping was performed in 42 patients with mild, moderate, or great complexity congenital heart disease. Acute success was 100%, with no complications and minimal recurrence over 48 months. This approach eliminates radiation exposure while maintaining safety and efficacy across select yet diverse CHD anatomies.

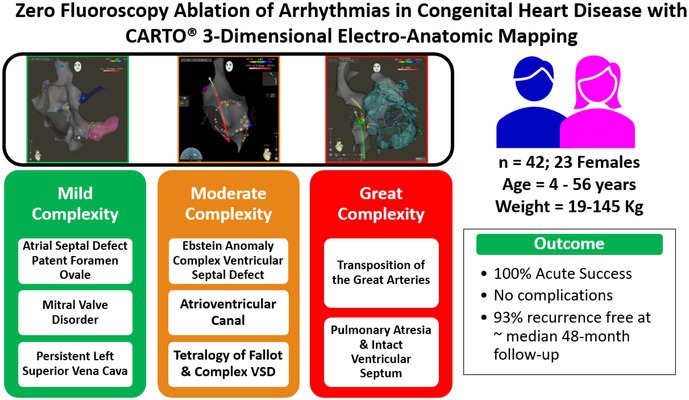

## 1 Introduction

Arrhythmias are common in patients with congenital heart disease (CHD), who often require multiple catheterization procedures throughout their lifetimes [1, 2]. Fluoroscopy has traditionally been used to guide catheter placement during ablations, but it carries risks of radiation exposure including dermatitis, cataracts, thyroid dysfunction, teratogenic effects, and malignancy [1, 2]. The risk is not only for patients, but also for the medical staff in the electrophysiology laboratory [3, 4]. Techniques for radiation reduction or elimination have advanced with the use of three‐dimensional electroanatomic mapping (3D EAM) systems, allowing for zero‐fluoroscopy catheter ablation in both structurally normal hearts and in CHD patients [5, 6]. CHD patients, in particular, stand to benefit from zero‐fluoroscopy approaches due to their increased cumulative exposure to medical radiation [5, 7]. However, published experience with zero‐fluoroscopy ablation in CHD has been limited to small case series [6, 8, 9, 10]. We report our single‐center experience using a CARTO 3D EAM (Biosense Webster, Diamond Bar, CA) system to perform arrhythmia ablations without fluoroscopy in children and adults with CHD.

## 2 Methods

### 2.1 Study Population and Data Collection

We conducted a retrospective review of all patients with CHD who underwent catheter ablation of arrhythmia without fluoroscopy at our institution from November 2016 through January 2025. Patient data including CHD diagnosis, arrhythmia type, procedural details, outcomes and recurrence were collected. CHD complexity was classified according to ACC/AHA guidelines into mild, moderate or great complexity categories [11]. All procedures were performed by a single electrophysiologist in a pediatric/congenital academic cardiology practice. In all cases, electroanatomic mapping was performed with the CARTO system. No lead aprons were worn by the primary operators, confirming commitment to zero‐fluoroscopy technique. All the procedures were performed under general anesthesia and endotracheal intubation.

### 2.2 Vascular Access

Vascular ultrasound guidance was utilized to obtain vascular access. In patients weighing less than 30 kg, three femoral venous sheaths were placed. In those weighing more than 30 kg, four femoral venous sheaths were placed to facilitate antegrade access to the right heart or to the left heart via an inter‐atrial communication or trans‐baffle approach to the pulmonary venous atrium in patients with D Transposition of the great arteries (D‐TGA) repaired with atrial switch operation. Femoral arterial access was obtained in patients with ventricular tachycardia to allow retrograde access to the left ventricle or aortic root.

### 2.3 Catheter Placement

Catheters were advanced under CARTO 3D EAM guidance. A deflectable decapolar catheter (DecaNav, Biosense Webster) was advanced via the femoral venous access to create the shell of the inferior vena cava (IVC), right atrium (RA), delineating the superior vena cava (SVC), coronary sinus (SC), and the tricuspid valve (TV) annulus. In patients with D‐TGA and atrial switch operation, the systemic venous baffle was delineated with creation of the shell. Upon creation of the shell, standard catheter placements included His bundle (CRD2, Abbott Technologies), right ventricular (RV) (Josephson 2 Abbott Technologies), and the deflectable DecaNav catheter was left engaged in the CS; in patients under 30 kg, a single Webster deflectable octapolar catheter (Biosense Webster) was used to span the right atrium/His bundle to right ventricle in lieu of separate His/ventricular catheters. Radiofrequency (RF) ablation catheters were chosen based on patient weight and anatomy. Non‐irrigated 4 mm tip catheters (Navistar, Johnson & Johnson/Biosense Webster) in various curve configurations (B, D or F curve) were utilized, with irrigated tip catheters (3.5 mm Thermacool, Johnson & Johnson/Biosense Webster) reserved for select cases (e.g., adult patients or ventricular arrhythmias). A CARTO‐created 3D shell of the RA (and LA in those with an interatrial communication) and as indicated by arrhythmia mechanism right ventricle, left ventricle or aorta was used for navigation instead of fluoroscopy.

### 2.4 Non‐Fluoroscopic Sheath Exchange

**FIGURE 1:**
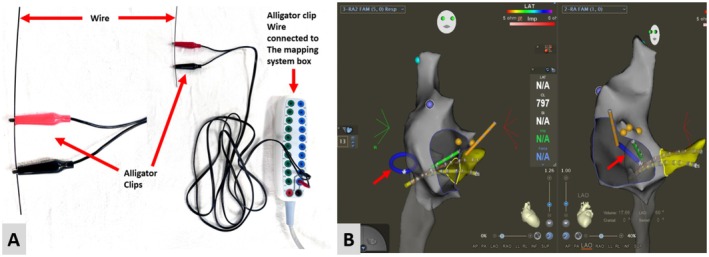
Panel A demonstrates the assembly of alligator clips attached to the guidewire, and cables connecting the alligator clips with 3D mapping box. Panel B demonstrates the right atrial shell with catheters in position. The guidewire utilized for sheath exchange was visualized (red arrows) in the heart by attaching alligator clips and connecting it with the mapping system for sheath exchange.

Long non‐steerable sheaths (Abbott St. Jude Medical: Fast‐Cath Sept Guiding Introducer Sheath SEPT or Abbott SRO Fast‐Cath Guiding Introduced Swartz) were employed for catheter stability when necessary. The right femoral venous access sheath was utilized for sheath exchange. Sterile alligator clips with connected cables were connected to the flexible long sheath exchange wire. The cables were then connected to the CARTO 3D EAM mapping system. The wire was then advanced under EAM guidance via the previously placed short femoral venous sheath and positioned in the atrium (Figure 1 and Video 1). The pre‐existing short femoral venous sheath was removed and replaced with a non‐steerable long sheath, which was advanced over the wire positioned in the right atrium. The wire and the dilator were then withdrawn leaving the sheath in place for mapping and ablation.

**VIDEO 1:** The video demonstrates the non fluoroscopic advancement of a guidewire within the heart using the electroanatomic mapping system. Video content can be viewed at https://onlinelibrary.wiley.com/doi/10.1002/joa3.70225.

### 2.5 Intracardiac Echocardiography (ICE)

The ICE CARTOSOUND module (Biosense Webster, Diamond Bar, CA, USA) was employed in selected cases, including two patients with severe Ebstein anomaly to delineate the true tricuspid annulus; patients with D‐TGA following atrial switch repair to define the systemic and pulmonary venous baffles and TV annulus; and a patient with ventricular tachycardia to delineate the right and left ventricular outflow tract and coronary cusps, thereby facilitating targeted ablation.

### 2.6 Mapping and Ablation Catheters

Mapping was performed using a DecaNav catheter (Biosense Webster), a radiofrequency (RF) ablation catheter (Navistar or ThermoCool), or high‐density mapping catheters such as PentaRay or OctaRay, as dictated by the arrhythmia substrate for complex or focal atrial arrhythmias. For patients with typical supraventricular tachycardia (e.g., atrioventricular nodal reentrant tachycardia [AVNRT] or Wolff Parkinson White [WPW] syndrome), non‐irrigated tip RF ablation catheters were used. In cases of ventricular tachycardia (VT), atrial flutter, intra‐atrial reentrant tachycardia (IART), or focal atrial tachycardia, irrigated‐tip 3.5‐mm ThermoCool RF ablation catheters were employed.

Acute procedural success was defined as successful ablation of the targeted arrhythmia mechanism with non‐inducibility of that arrhythmia post‐ablation.

## 3 Results

### 3.1 Patient Characteristics

A total of 42 patients with CHD underwent zero‐fluoroscopy ablation of arrhythmia substrate during the study period (Table 1). Descriptive statistics and central tendency were employed to summarize the findings due to the cohort size. Table 1 details the patient demographics, anti‐arrhythmic medication use pre‐ablation and the cohort within each CHD complexity. CHD complexity and associated arrhythmia mechanisms are detailed in Table 2.

**TABLE 1:** Patient characteristics.

Patient characteristics	Values
Median age (years)	14 (4–56)
Median weight (kg)	55 (19–145)
Median height (cm)	160 (112–189)
Median BSA (m^2^)	1.6 (0.74–2.67)
Female sex	23 (55%)
On antiarrhythmic medication pre‐ablation	27 (64%)
Congenital heart disease complexity	Mild (*n* = 21); moderate (*n* = 16); great (5)

**TABLE 2:** Classification of congenital heart disease (CHD) complexity and arrhythmia substrate.

Complexity	Condition	Number of patients	Arrhythmia mechanism
Mild complexity CHD (*n* = 21)	Secundum atrial septal defect (ASD)	3 (2 large, 1 small)	AVNRT
	Fenestrated ASD	1	ORT‐WPW
	Sinus venosus ASD	1	FAT
	Patent foramen ovale (PFO) requiring LA ablation	11	ORT, FAT
	ASD + persistent LSVC	2	AVNRT, ORT
	Persistent LSVC (isolated)	1	ORT‐WPW
	Mitral valve prolapse	1	VT
	Congenital aortic valve stenosis/insufficiency	1	AVNRT, FAT
Moderate complexity CHD (*n* = 16)	Ebstein anomaly (unrepaired)	6	AVNRT, WPW, ORT
	Ebstein anomaly (repaired – cone procedure or tricuspid valvuloplasty)	2	ORT‐WPW, AVNRT, FAT
	Repaired partial AV canal defect and superior sinus venosus ASD	3	AFL, FAT
	Pulmonary stenosis (post‐surgical valvotomy)	1	ORT‐WPW
	Repaired tetralogy of fallot (TOF)	3	AFL, FAT
	Ventricular septal defect (VSD) with anomalous coronary artery	1	VT
Great complexity CHD (*n* = 5)	D‐transposition of great arteries (D‐TGA) post arterial switch	1	AVNRT
	D‐TGA post mustard (atrial switch)	2	AFL, FAT
	L‐transposition of great arteries (L‐TGA)	1	FAT
	Pulmonary atresia with intact ventricular septum (PA‐IVS) post RVOT reconstruction	1	AFL

Abbreviations: AFL, atrial flutter; AVNRT, AV node reentry tachycardia; CHD, congenital heart disease; FAT, focal atrial tachycardia; ORT, orthodromic reciprocating tachycardia; VT, ventricular tachycardia; WPW, Wolff Parkinson White syndrome.

### 3.2 Electrophysiologic Study

During EPS, incremental pacing and programmed stimulation were used to induce arrhythmias. In patients with pre‐excitation, accessory pathway conduction was assessed, and entrainment confirmed macro‐reentrant mechanisms. Three‐dimensional mapping enabled localization of critical isthmuses and pathways without fluoroscopy, with high‐density catheters (PentaRay/OctaRay) used selectively. Ablation catheter curves were chosen by patient anatomy (D‐curve 57%, B‐curve 33%, F‐curve 10%); in two Ebstein patients, larger curves were required. Irrigated catheters were used in 5 adult/VT cases. Four diagnostic catheters were used in patients > 30 kg and three in smaller patients. Long non‐ steerable sheaths (Abbott St. Jude Medical: Fast‐Cath Sept Guiding Introducer Sheath SEPT or Abbott SRO Fast‐Cath Guiding Introduced Swartz) for catheter stability were required in 9 patients (21%), most commonly in those with complex anatomy such as Ebstein anomaly, those with difficult transseptal access or anatomy (e.g., D‐TGA Mustard, ASD + PLSVC), and in one patient with a repaired AV canal. In the remaining ~79% of cases, catheter stability was achieved without long sheaths by utilizing the 3D mapping guidance and careful catheter selection.

### 3.3 Arrhythmia Mechanism

Arrhythmias included AVNRT (14%), manifest WPW (21%), concealed AVRT (26%), AFL (12%), AT (14%), and VT (7%). Some patients had multiple mechanisms. Table 2 summarizes arrhythmia type by CHD complexity.

### 3.4 Arrhythmia Substrate Ablation

In our cohort, the tachycardia focus was located in the right atrium in 25 patients (60%) and in the left atrium in 17 patients (40%). All left‐sided arrhythmias (left‐sided accessory pathways or left atrial tachycardias) were approached via the patent foramen ovale (PFO) access. Patients with an ASD or PFO (*n* = 18) provided a ready route for LA access. ICE was utilized in total 5 patients; to demonstrate the systemic venous baffle in patients with D‐TGA atrial switch, tricuspid valve annulus in Ebstein anomaly and right and left ventricular outflow tract with aortic sinuses for VT ablation. The median number of RF applications delivered was 14 (range 1–38). Acute procedural success‐defined as complete non‐inducibility of the targeted arrhythmia and elimination of accessory pathway conduction if applicable‐was achieved in all 42 patients. There were no acute complications such as AV block, vascular injury, or thromboembolism.

### 3.5 Specific Congenital Heart Defect Subgroups

#### 3.5.1 Mild Complexity CHD

- ASD/PFO: Eleven patients had an ASD or a PFO that provided direct access to the left atrium for ablation of left‐sided pathways or arrhythmias. In these cases, the septal defect obviated the need for transseptal puncture. A long sheath was used in one patient to enhance catheter stability within the left atrium. All left‐sided arrhythmias in this subgroup were successfully ablated without the use of fluoroscopy.
- ASD with persistent LSVC: Two patients had a combination of a secundum atrial septal defect (ASD) and a persistent left superior vena cava (LSVC) draining into the coronary sinus (CS), with atrioventricular nodal reentrant tachycardia (AVNRT) as the presenting arrhythmia. The dilated CS resulting from the LSVC elevated the floor of Koch's triangle. Using CARTO guidance, slow‐pathway ablation was successfully performed by targeting a slightly more superior region of Koch's triangle than usual. Both procedures achieved acute success without the use of fluoroscopy.

#### 3.5.2 Moderate Complexity CHD

**FIGURE 2:**
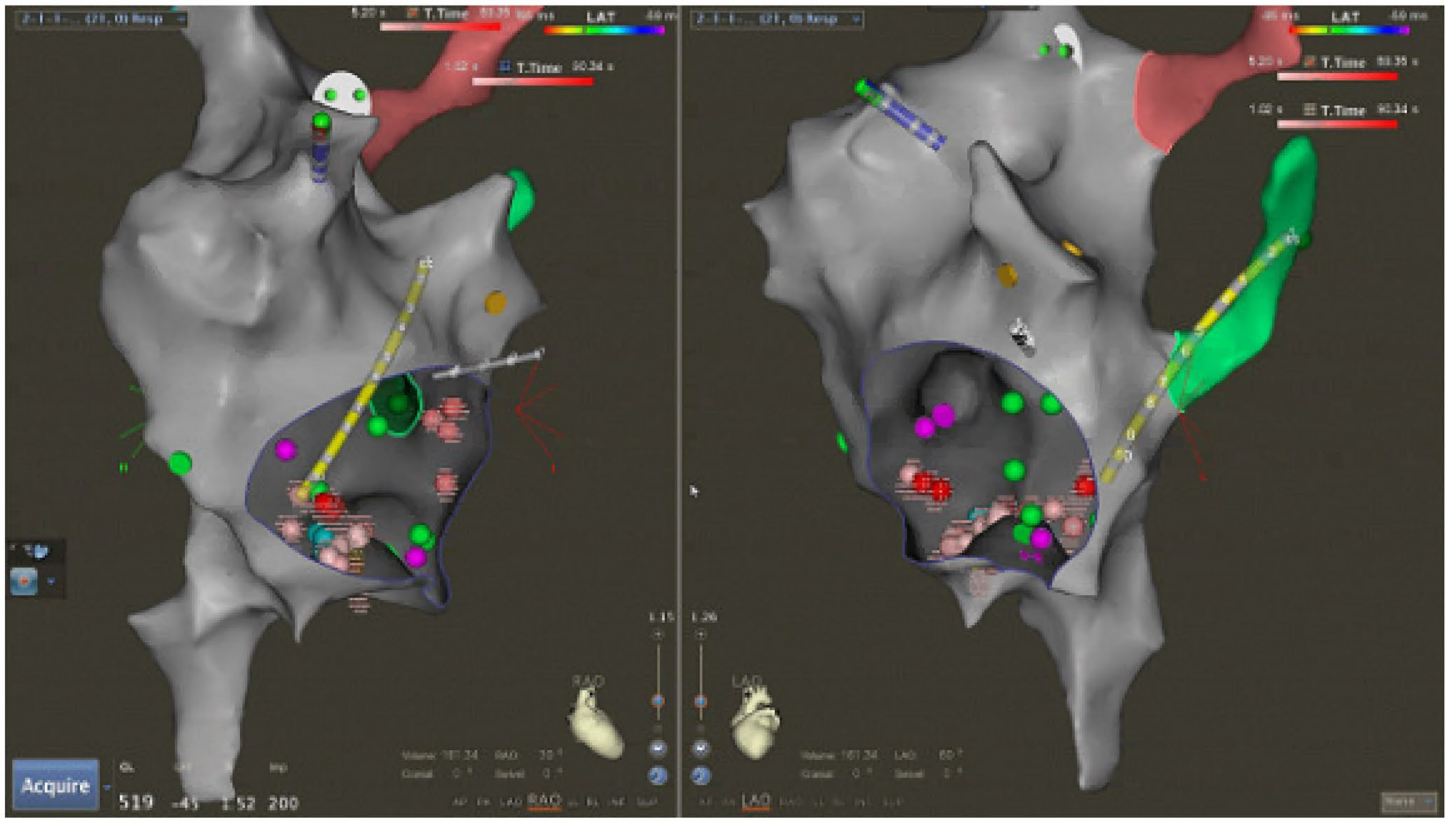
Patient with Ebstein anomaly of the tricuspid valve successfully ablated for right lateral accessory pathway and AVNRT (red dots).

**FIGURE 3:**
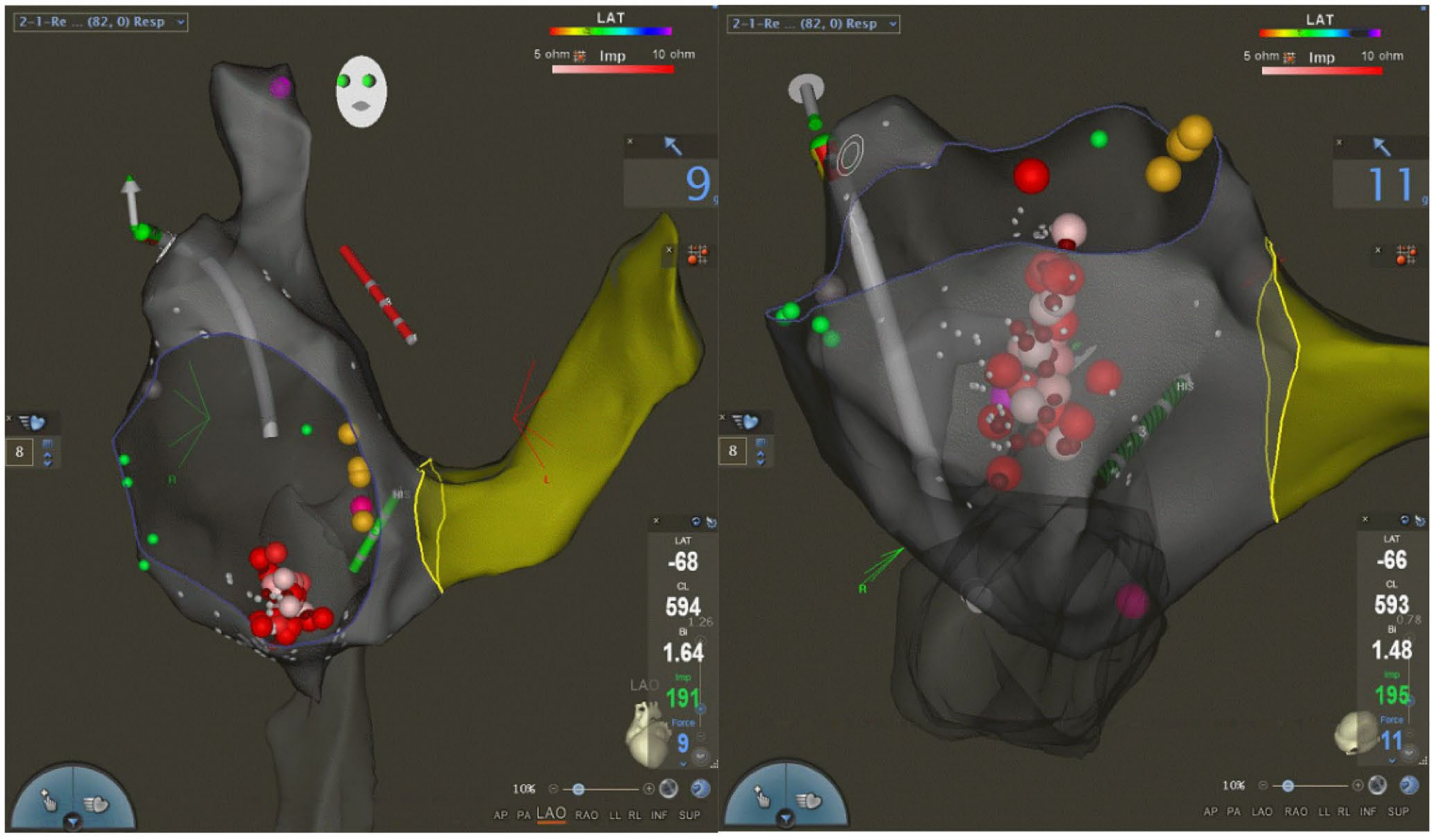
Patient with repaired AV canal defect and Isthmus dependent atrial flutter. Red/pink dots represent RF line of successful ablation. Note posteriorly displaced AV node and His signals (dark pink dot) near the mouth of coronary sinus. Ablation was targeted at a slightly lateral location from usual.

- Ebstein anomaly: In this series, eight patients had Ebstein anomaly (6 unrepaired, 2 post‐surgical repair). As expected, accessory pathways were common in this group (7 of 8 had at least one accessory pathway, and 5 had WPW syndrome). We ablated a total of 9 distinct accessory pathways in these 8 patients (locations ranged from right posteroseptal to right posterolateral). ORT was noted in 6 of these patients with manifest WPW, whereas one repaired Ebstein patient had ORT via a concealed accessory pathway. All arrhythmias in Ebstein patients were successfully eliminated. One unrepaired Ebstein patient had two pathways ablated (both a right posterior septal and a right posterolateral pathway); the manifest posterolateral pathway recurred on ECG months later, but without clinical SVT, and thus has been managed medically. Another severe Ebstein patient had both a manifest right posterolateral pathway and AVNRT; both were ablated without complication aided by ICE to define the true TV annulus (Figure 2).
- Repaired AV canal: Two adult female patients with repaired partial atrioventricular (AV) canal defects underwent ablation. One had typical cavotricuspid isthmus‐ dependent atrial flutter, and the other had focal right atrial tachycardia. Both arrhythmias were successfully ablated without the use of fluoroscopy (Figure 3).
- Repaired TOF: Three patients with repaired TOF (ages 27, 41, and 57) underwent ablation without fluoroscopy. All had atrial tachyarrhythmias. Two had typical isthmus‐dependent atrial flutter (one of whom also had a superior‐crista terminalis origin atrial tachycardia), and one had a focal AT near the atriotomy patch. Successful ablation was achieved in all. Because patients with TOF have a predisposition to macro‐reentrant atrial circuits, we routinely placed multipolar catheters to map for latent flutter circuits. In one case, we employed dual crista catheters to ensure any high right atrial reentry would be detected. All identified arrhythmias in TOF were eliminated, and no patient developed new arrhythmias. There was no arrhythmia recurrence in any patient at follow‐up.

#### 3.5.3 Great Complexity CHD

**FIGURE 4:**
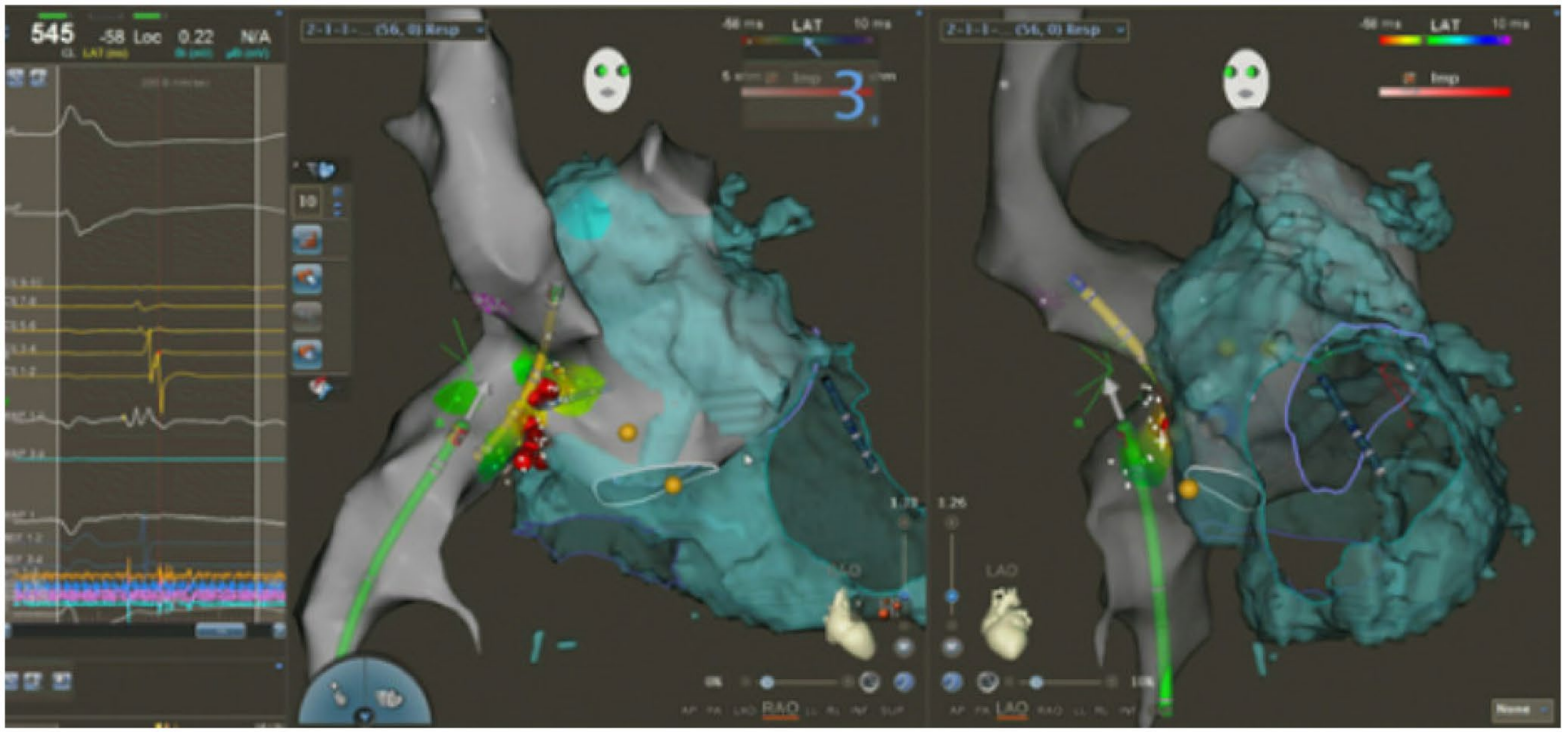
Patient with mustard operation for D‐transposition of the great arteries, successful ablation across the baffle for atrial flutter, aided by intracardiac echocardiography.

- D‐TGA: Three patients had D‐TGA; one status post arterial switch operation and two status post Mustard atrial switch. The arterial switch patient (31‐year‐old male) had atypical atrioventricular nodal reentrant tachycardia (AVNRT), which was successfully treated with slow‐pathway modification in the basal third of Koch's triangle. The two Mustard‐repaired patients presented with atrial tachyarrhythmias involving the systemic venous baffle. CARTO mapping delineated the baffle anatomy and identified reentrant circuits near the baffle‐native atrium junction. Ablation was successful in both cases without fluoroscopy. In one patient, focal atrial tachycardia near the right upper pulmonary vein baffle insertion was ablated using trans‐baffle access guided by 3D EAM and ICE. The CARTOSOUND catheter positioned in the systemic venous baffle revealed a small baffle leak at the superior limb of the SVC baffle, which was marked on the EAM map. The ablation catheter was then advanced across the leak to successfully target the focal atrial tachycardia (Figure 4).
- Other complex lesions: One patient with pulmonary atresia and intact ventricular septum (PA‐IVS) status post repair with right ventricle—to ‐pulmonary artery conduit developed a Wolff Parkinson White (WPW) pattern due to a right lateral accessory pathway, which was successfully ablated without fluoroscopy. Another patient with congenitally corrected transposition of the great arteries (L‐TGA) had atrioventricular nodal reentrant tachycardia (AVNRT), which was ablated by carefully mapping the inverted septal anatomy, accounting for the mirror‐image orientation of the atrioventricular node and slow pathway region.

### 3.6 Ventricular Arrhythmia Ablation

**FIGURE 5:**
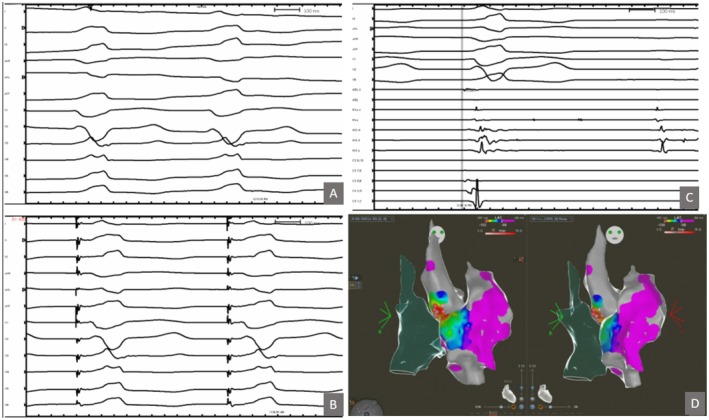
Patient with ventricular septal defect and ventricular tachycardia focus in the coronary cusp. Intracardiac echocardiography was utilized to delineate the coronary cusps in this patient. (A) Clinical VT; (B) 12/12 pace‐map at the junction of Non and Right coronary cusp; (C) Mapping in the coronary cusp demonstrating earlier activation than the His bundle location; (D) 3D Electro‐Anatomic map demonstrating the location of successful RF applications (red dots).

**FIGURE 6:**
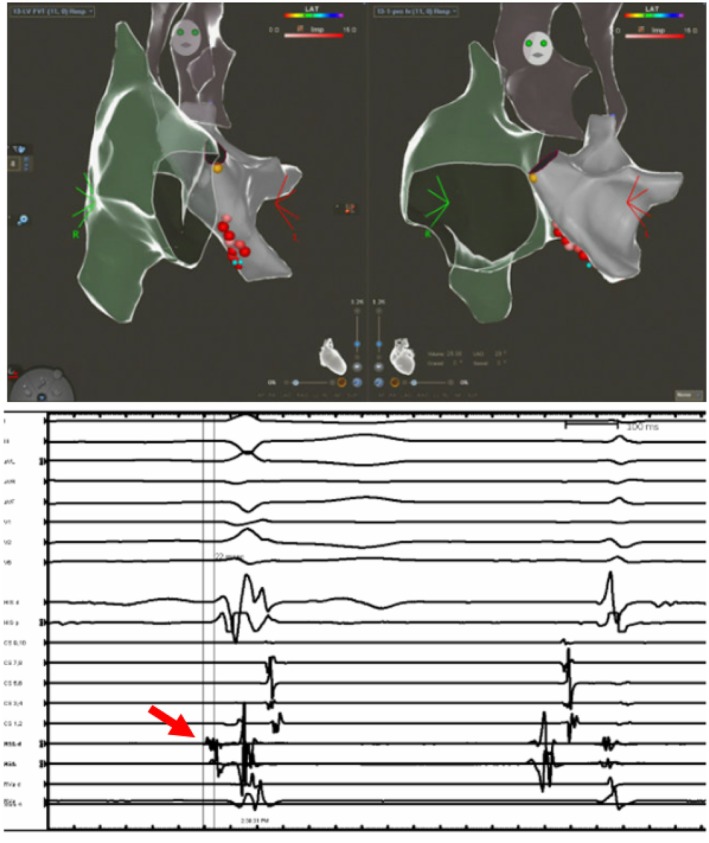
Patient with fascicular VT, electro‐anatomic map was created with retro aortic approach delineating the right atrial (green shell), aorta (dark gray shell) and the left ventricle light gray shell. Red dots represent the site of successful radiofrequency applications. The electrogram, demonstrates the Purkinje potential at the site of ablation during a ventricular beat.

One patient with a mid‐muscular VSD and anomalous coronary artery origin had VT from the RVOT and non‐coronary cusp. Using 3D mapping with CARTOSOUND and ICE guidance, the VT focus near the commissure of the non‐coronary and right coronary cusps was successfully ablated without fluoroscopy. Another patient with congenital mitral valve abnormality and mitral valve prolapse had a fascicular left ventricular VT that was mapped and ablated with the ablation catheter guided by 3D EAM (Figures 5 and 6).

### 3.7 Follow‐Up

The median follow‐up duration of 48 months (range 2–99 months), there were three recurrences of the original arrhythmia. Specifically, one patient with Ebstein anomaly and a WPW pathway had return of conduction in a right lateral accessory pathway approximately 6 months post‐ablation; this patient's recurrent pre‐excitation (WPW) has been managed conservatively to date, as no symptomatic SVT has recurred. Another patient with a large ASD and PLSVC (concealed left‐sided accessory pathway) had recurrence of ORT at 1‐year post‐ablation and was successfully treated with beta‐blocker therapy per the family's preference. A third patient (with multiple arrhythmias initially ablated) had recurrence of an SVT at 1 month, that was successfully ablated again without fluoroscopy and there was no recurrence thereafter. In two patients, non‐sustained arrhythmias were noted on follow‐up monitoring but did not require intervention: one repaired TOF patient had occasional brief runs of atrial tachyarrhythmia with varying P‐wave morphology which have been managed conservatively, and one patient with Ebstein anomaly had non‐sustained junctional tachycardia early in recovery that resolved spontaneously. There were no late complications observed related to the zero‐fluoroscopy approach.

One patient with a VSD and ablation involving RVOT, had successful ablation, however he had recurrence with a different morphology at follow‐up, that was successfully ablation in the coronary cusp (non and right coronary cusp junction) with no further recurrence.

## 4 Discussion

Prior reports in small series have demonstrated the feasibility of NFA in patients with CHD [8, 10]. This study demonstrates the feasibility of performing zero‐fluoroscopy RF ablation of arrhythmia in patients with mild, moderate and great complexity CHD using the CARTO 3D EAM system. Combination of UNIVU with CARTO 3D EAM system has been demonstrated to aid fluoroscopy reduction for various types of arrhythmias, however this was not available at our institution [10]. We report a high acute success rate (100%) in our cohort of 42 patients, with no acute complications and minimal recurrences. Our experience expands upon prior small series by including a broader range of congenital heart defects (from simple ASDs and PFOs to repaired TOF, Ebstein anomaly and atrial switch operated D‐TGA patients) and arrhythmia substrates (including AVNRT, WPW/AVRT, AFL, AT, and even some VT). We have demonstrated that with careful planning and technique adjustments, fluoroscopy can be eliminated even in some moderate and great complexity CHD cases. A prior study has demonstrated NFA is patients with moderate and some great complexity patients as well [10].

Our results provided insight into several CHD subgroups. In patients with mild complexity CHD, the ablation procedure was a straightforward exercise. Patients with persistent left superior vena cava may have a displaced slow pathway or His bundle location [12]. In our series, the successful applications were required to be placed at a slightly higher up location in the tringle of Koch.

Three predominant CHD in moderate complexity group included, Ebstein anomaly, repaired AV canal defect and repaired TOF. Ebstein anomaly of the tricuspid valve represents failure of delamination of the septal leaflet of the tricuspid valve and discrepancy between the tricuspid valve leaflets and true annulus [13]. Patients with Ebstein anomaly may have a multitude of arrhythmias with accessory pathway mediated arrhythmias being the most common [13, 14]. Outcomes for AP ablation in patients with EA have improved, but there is still a relatively high recurrence risk requiring repeat procedures [14]. ICE with CARTOSOUND may help delineate the true tricuspid valve annulus and facilitate successful ablation [13, 15, 16]. All arrhythmias in Ebstein patients were successfully eliminated and the procedure was aided with ICE CARTOSOUND module.

In patients with repaired AV canal defects, atrial arrhythmias are frequently encountered with advancing age [17]. The AV node may be more posteriorly displaced in patients with AV canal defect [18]. When performing AV node slow pathway modification, or typical flutter ablation, care must be taken to carefully delineate the His bundle signals and stay more lateral on the isthmus to avoid the high‐risk region for AV block. Both atrial and ventricular arrhythmias are common after repaired TOF and the risk goes up with advancing age [19]. Because patients with TOF have a predisposition to macro‐reentrant atrial circuits, multipolar catheters to map for latent flutter circuits are beneficial. White ventricular arrhythmias may also occur in patients with TOF, our series did not have any patients with VT. ICE may facilitate identification of anatomic isthmuses and aid ablation of ventricular tachycardia in TOF [20].

Great complexity CHD cohort included D‐TGA after atrial switch operation, congenitally corrected TGA and PA‐IVS. Atrial arrhythmias are common among D‐TGA patients repaired with an atrial switch operation. Often, the arrhythmia source may be across the other side of baffle, making ablation access a challenge. It is therefore critical to recognize the underlying anatomy [21, 22, 23]. In addition to atrial arrhythmias, AVNRT may be encountered in patients with D‐TGA and ablation strategies may need to be individualized [24]. Typical atrial flutter ablation may require a trans‐baffle or retro‐aortic approach for creating caval‐tricuspid isthmus block, which can be aided with ICE [23].

Our cases underscore that even intra‐atrial reentrant tachycardias in atrial switch anatomy can be tackled with modern mapping systems without fluoroscopy.

Ablation of ventricular arrhythmia may be successfully accomplished without fluoroscopy in RVOT and in patients with structural heart abnormality [25, 26]. ICE with CARTOSOUND facilitated NFA by creating the RVOT anatomy, coronary cusp anatomy. Cases of zero fluoroscopy ablation of fascicular VT have also been previously reported [27]. We were able to accomplish this in one patient with congenital mitral valve abnormality and mitral valve prolapse. NFA was facilitated with mapping performed along the LV septum as guided by EAM.

Several factors facilitated the consistent use of a zero‐fluoroscopy approach in our series. Vascular access was obtained with ultrasound guidance, with the number of sheaths tailored to patient size. Catheter placement was guided entirely by 3D EAM, with shells of the relevant chambers created for navigation, and sheath exchanges were performed under mapping guidance using the alligator clip technique (Figure 1 and Video 1). ICE was selectively employed in complex cases, such as Ebstein anomaly and D‐TGA, to further define anatomic landmarks.

These strategies allowed all procedures to be completed without emergent fluoroscopy. The CARTO system reliably guided catheter navigation within baffles or patch materials by using electroanatomic landmarks and reconstructed geometry. For example, atrial baffles in Mustard/Senning repairs were delineated to enable successful ablation, while CARTO mapping of the dilated right atrium in Ebstein anomaly identified the true AV groove, with ICE confirmation in cases of markedly distorted anatomy. Long non‐steerable sheaths were required in approximately 21% of patients with challenging anatomy; however, in most cases, stability was achieved with mapping guidance and tailored catheter selection.

Together, these techniques demonstrate that proficiency in 3D mapping, selective use of ICE, and careful catheter strategy can eliminate the need for fluoroscopy, even in complex CHD, without compromising outcomes. Acute success and recurrence rates were comparable to conventional fluoroscopy‐guided approaches, and no case in our series required bailout X‐ray.

### 4.1 Limitations

This study is a single‐center retrospective analysis with a relatively modest sample size (42 patients), which may limit generalizability. All procedures were performed by a single experienced operator, which may introduce operator‐specific bias and may not reflect the learning curve that other centers would experience. We did not include patients with some forms of great complexity CHD or those with certain anatomies (e.g., Fontan circulation) in this series, so our results cannot be directly extrapolated to those entire groups without further studies.

## 5 Conclusion

In summary, zero‐fluoroscopy ablation of various types of arrhythmias was successfully and safely accomplished using the CARTO 3D EAM system in a cohort of 42 patients with mild, moderate and great complexity CHD. Acute outcomes were equivalent to those reported with traditional fluoroscopic techniques, and no procedure‐related complications were encountered. Our findings support that a non‐fluoroscopic approach to ablation should be considered in CHD patients with suitable anatomy and arrhythmias. With growing experience and technological advancements, zero‐fluoroscopy techniques could potentially be expanded to an even broader range of congenital substrates and arrhythmia types, further reducing radiation exposure for this vulnerable patient population. Multi‐center studies and continued innovation will further define the role of zero‐fluoroscopy ablation in CHD and help establish best practices for widespread adoption.

## Author Contributions

Shailendra Upadhyay: conceived and designed the study, performed all ablation procedures, interpreted data, supervised manuscript development, and served as lead author for manuscript drafting and revisions. Jenna Schermerhorn: contributed to data interpretation, literature review, and critical manuscript revisions. Whitney Fairchild: assisted with data collection, patient follow‐up, and contributed to the methods and results sections. Jamie Bopp: supported data acquisition, procedural logistics, and patient tracking. Irfan Warsy: provided oversight for study design, contributed to data interpretation, and critically revised the manuscript for intellectual content.

## Ethics Statement

Not applicable, the study was approved by our institutional IRB. As a retrospective study, individual informed consent was waived.

## Conflicts of Interest

The authors declare no conflicts of interest.

## Data Availability

The data supporting the findings of this study are available from the corresponding author upon reasonable request.
